# Colloidal and Biological Characterization of Dual Drug-Loaded Smart Micellar Systems

**DOI:** 10.3390/polym16091189

**Published:** 2024-04-24

**Authors:** Hildegard Herman, Delia M. Rata, Anca N. Cadinoiu, Leonard I. Atanase, Anca Hermenean

**Affiliations:** 1 “Aurel Ardelean” Institute of Life Sciences, Vasile Goldis Western University, Rebreanu Street, No. 86, 310414 Arad, Romania; hildegard.i.herman@gmail.com (H.H.); anca.hermenean@gmail.com (A.H.); 2 Faculty of Medicine, “Apollonia” University of Iasi, Pacurari Street, No. 11, 700511 Iasi, Romania; iureadeliamihaela@yahoo.com (D.M.R.); jancaniculina@yahoo.com (A.N.C.); 3 Academy of Romanian Scientists, 050045 Bucharest, Romania

**Keywords:** pH-sensitive polymeric micelles, paclitaxel, ursolic acid, poly(2-vinyl pyridine)-b-poly(ethylene oxide), drug delivery system

## Abstract

Smart polymeric micelles (PMs) are of great interest in drug delivery owing to their low critical micellar concentration and sizes. In the present study, two different pH-sensitive poly(2-vinyl pyridine)-b-poly(ethylene oxide) (P2VP-b-PEO) copolymer samples were used for the encapsulation of paclitaxel (PTX), ursolic acid (UA), and dual loading of PTX and UA. Based on the molecular features of copolymers, spherical PMs with sizes of around 35 nm and 140 nm were obtained by dialysis for P2VP_55_-b-PEO_284_ and P2VP_274_-b-PEO_1406_ samples, respectively. The micellar sizes increased after loading of both drugs. Moreover, drug encapsulation and loading efficiencies varied from 53 to 94% and from 3.2 to 18.7% as a function of the copolymer/drug ratio, molar mass of copolymer sample, and drug type. By FT-IR spectroscopy, it was possible to demonstrate the drug loading and the presence of some interactions between the polymer matrix and loaded drugs. In vitro viability was studied on 4T1 mammary carcinoma mouse cells as a function of time and concentration of drug-loaded PMs. UA-PMs and free PMs alone were not effective in inhibiting the tumor cell growth whereas a viability of 40% was determined for cells treated with both PTX- and PTX/UA-loaded PMs. A synergic effect was noticed for PTX/UA-loaded PMs.

## 1. Introduction

In recent decades, important progress has made by scientists in the fight against cancer by developing new anti-tumor drug molecules. Nevertheless, the proposed strategies remain aggressive and painful due to the high toxicity of these drugs. Moreover, these molecules can precipitate upon administration by intravenous injection leading to the formation of aggregates, which might cause other medical complications [1]. To increase the water solubility, but also to assure a controlled and sustained drug release, numerous nanocarriers were created [2]. Advantages of these drug delivery systems (DDSs) are (i) protection of the active pharmaceutical ingredient (API) from possible undesired degradation and quick metabolism; (ii) transport to the desired site; and (iii) increase in patient compliance owing to the reduction of toxicity by a less frequent administration [3]. 

Among the studied DDSs, polymeric micelles (PMs) have shown promising advantages and have undergone significant development in recent years [4]. PMs, with nanometric sizes, are obtained by the self-assembly of amphiphilic graft or block copolymers, of suitable composition and molar mass, in a core-shell or corona structure [5]. In this case, hydrophobic APIs can be solubilized in the micellar core whereas the hydrophilic corona stabilizes PMs, protects APIs, and can improve their biocompatibility. In addition, the micellar corona can provide the stealth effect, which means that the PMs are invisible to the immune system after administration. FDA-approved poly(ethylene glycol) (PEG) is one of the hydrophilic polymers that inhibits the adsorption of plasma proteins, the first link in the immune system, on the surface of micelles, by steric repulsions. Thus, recognition by the organism is avoided, and their blood circulation time is prolonged in order to be able to reach organs other than the liver [6]. The nanometric sizes of PMs are another serious advantage as they allow accumulation in highly vascularized areas, such as tumors, by the enhanced permeability and retention (EPR) effect [7,8].

These amphiphilic copolymers are also characterized by a critical micellar concentration (C.M.C.) having, generally, values in the micromolar or even nanomolar range, which can assure them a very good stability when diluted in the blood. C.M.C. decreases when the length of the hydrophobic block increases [9]. In this case, the volume of the micellar core increases, and thus the encapsulation efficiency also increases. The encapsulation efficiency can also be improved by changing the nature of the hydrophobic block in order to optimize the compatibility with the loaded API [10].

Due to these features, the release in a controlled and sustained manner of the loaded API can be difficult to induce. In this context, the so-called “smart” copolymers have attracted interest as their physicochemical properties can be modulated in response to the modification of a specific external stimulus. In order to increase the efficiency of PMs as DDSs, pH- and thermo-sensitive copolymers are extensively studied as these stimuli can be easily modified to trigger the API release at specific conditions [5,11,12]. This modification, which can be physical or chemical in nature, is most often reversible.

Due to the different pH values encountered in blood, organs, or tumor cells, numerous studies focused on pH-sensitive micellar systems with application in the biomedical field [13,14,15,16,17]. A pH-sensitive polymer has a function capable of protonation or deprotonation as a function of pH. A change in the pH values induces the modification of the degree of dissociation of an acid to a specific value called pKa. Thus, this variation affects hydrogen bonds, as well as ionic and hydrophobic interactions, and therefore modifies the hydrophilic/hydrophobic balance, which affects the solubility of the polymer in an aqueous medium (the polymer chains are contracted or extended) generating the disorganization of PMs into unimers or modifying their morphology. As a result, the chains of a polyacid (e.g., poly(acrylic acid) (PAA) with pKa~5) will precipitate at basic pH due to deprotonation [18,19]. On the contrary, a polybase (e.g., Poly(2-(dimethylamino)ethyl methacrylate) (PDMAEMA) with pKa~7.5) placed in an acidic environment will accept protons becoming protonated, and therefore the polymer chains will extend [16]. 

The micellization of pH-sensitive polymers based on di- and triblock poly(2-vinyl pyridine)-b-poly(ethylene oxide) (P2VP-b-PEO) copolymers was already studied as a function of different parameters [20,21]. It appeared that at low pH values, only unimers are present whereas at pH values higher than 5, self-assembly occurs leading to frozen-in micelles with a P2VP core and PEO corona. This pH-sensitive behavior can further modulate drug release [22]. Even if the physicochemical properties of these smart PMs are quite well studied, the translation to clinical studies is complicated because of the lack of complete in vitro and in vivo characterization. 

Paclitaxel (PTX) is a hydrophobic mitotic inhibitor with a powerful anti-cancer effect, against breast, ovarian, lung, head, and neck cancer. Because of water insolubility, PTX is commercially marketed in micellar solutions forming vehicle Cremophor EL to enhance drug solubility. However, the addition of Cremophor EL results in hypersensitivity, neurotoxicity, and altered pharmacokinetics of PTX. Currently, several strategies are in progress to develop a CrEL-free formulation of PTX, including biological approaches (oral administration), chemical approaches (prodrugs/analogs), and pharmaceutical approaches (use of co-solvents, emulsions, cyclodextrins, microspheres, and liposomes) [23,24]. PTX is widely used to treat breast cancer, one of the most common clinical cancers in the world, and the main cause of cancer-related death in women; however, its therapeutic efficacy is limited, due to the development of resistance. Thus, attempts to effectively reverse resistance are crucial for improving patients’ treatment options and prognoses.

Ursolic acid (UA) is a natural pentacyclic triterpene acid that demonstrated growth inhibition of many human cancer cell lines, such as breast cancer, gastric cancer, liver cancer, and skin cancer [25,26]. UA is considered as a potential chemotherapeutic agent suitable for cancer treatment. Additionally, in vitro research has shown that UA attenuates PTX resistance through multiple pathways [27,28]. Other studies showed the synergic effect of UA with cisplatin against cervical cancer cells [29] and PTX against human gastric carcinoma cells [30].

The aim of this study was to obtain dual drug-loaded PMs based on two P2VP-b-PEO copolymers, with different molar masses. PTX and UA were loaded separately but also together in order to study their synergic effect. After the colloidal characterization of the drug-loaded micellar systems, by TEM, DLS, and FT-IR, a detailed in vitro characterization was carried out. 

As our study is focused on breast cancer, the evaluation of the cytotoxic and pro-apoptotic effects of the PTX- and UA-loaded polymeric micelles was assessed in vitro by MTT assay on 4T1 mammary carcinoma mouse cells. The 4T1 mammary carcinoma mouse cell line has highly tumorigenic and invasive properties.

## 2. Materials and Methods

Paclitaxel was purchased from Thermo Scientific (Waltham, MA, USA), whereas ursolic acid was obtained from Sigma Aldrich (St. Louis, MO, USA). The 2VP and ethylene oxide were purchased from Fluka (Buchs, Switzerland). Phenylisopropyl potassium was synthesized starting from α-methylstyrene in the presence of methanol and perchloric acid during 48 h at 50 °C. 

Poly(2-vinylpyridine)-b-poly(ethylene oxide), P2VP-b-PEO, and diblock copolymers were synthesized by living anionic polymerization in THF, in the presence of phenylisopropyl potassium as an initiator [31,32]. The 2-Vinylpyridine was first polymerized at −75 °C for 2.5 h, ethylene oxide was cryo-distilled into the reactor, and the temperature was increased to 20 °C. The molecular properties of the copolymers are presented in Table 1.

**Figure 1:**
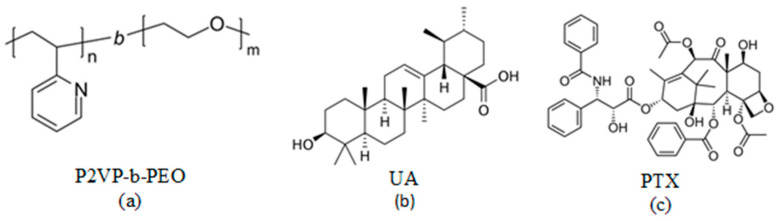
Chemical structures of P2VP-b-PEO copolymer (**a**), ursolic acid (**b**), and paclitaxel (**c**).

The chemical structures of the block copolymers and both drugs are provided in Figure 1.

### 2.1. Preparation of Free and Drug-Loaded Micellar Solutions

The preparation of nanomicellar systems was carried out by a dialysis method starting from a copolymer solution in dimethyl sulfoxide (DMSO). The solution was directly dialyzed, for 24 h, against 1 L of ultrapure water using cellulose dialysis membranes (molecular weight cut off: 12–14 kDa). Water was changed five times during the dialysis process. After this period, the micellar solution from the dialysis bag was collected, frozen, and then lyophilized in order to obtain a dry powder, which was stored at −4° before further use. The same procedure was used for the preparation of PTX- and ursolic acid-loaded micellar systems with the difference that different quantities of drugs were added to the solution of the block copolymer in DMSO, as shown in Table 2.

### 2.2. Physicochemical Characterization

The micellar sizes were determined by dynamic light scattering (DLS) measurements, which were carried out on a Malvern Nano-ZS Zetasizer (Malvern, UK) equipped with a 4 mW He–Ne laser operating at a wavelength of 532 nm and a scattering angle of 173°. The software package (ZS Xplorer version 3.3) of the instrument calculates, by using the Stokes–Einstein equation, the Z-average diameter, which is an intensity-weighted size average and the polydispersity index (PDI) of the sample. TEM microscopy was carried out with a Philips CM100 microscope equipped with an Olympus camera and transferred to a computer equipped with the Megaview system. The PMs’ aqueous suspension was displayed on a formvar-coated copper grid and analyzed after water evaporation. The interaction between the drugs and the copolymer were investigated by Fourier transform infrared spectroscopy (FT-IR) in absorbance mode within the domain of 400 to 4000 cm^−1^ using a Shimaszu IRSpirit spectrometer equipped with a QATR™-S Single-Reflection ATR Accessory.

### 2.3. Drug Encapsulation and Loading Efficiency (DEE and DLE)

In order to determine the encapsulation efficiency of both drugs, two calibration curves were constructed in DMSO, using different concentrations of drugs, and their absorbance values were recorded on a Nanodrop UV-Vis spectrophotometer (Nanodrop One UV-Vis Spectrophotometer, Thermo Fischer Scientific, Waltham, MA, USA). A known amount of the drug-loaded micelles, as powder, was dissolved in DMSO, and then these solutions were transferred to dialysis membranes. These membranes were immersed in DMSO, in Erlenmeyer flasks, under stirring in a water bath at 37 °C for 48 h in order to extract the active ingredients. The amount of both drugs from the micelles were spectrophotometrically quantified, based on their calibration curve, using a UV spectrometer. Drug encapsulation efficiency (DEE) and drug loading efficiency (DLE) were calculated using Equations (1) and (2), respectively:

(1) $$DEE\left(\%\right)=\frac{Amount\;of\;drug\;in\;micelles}{Amount\;of\;added\;drug}\times 100$$

(2) $$DLE\left(\%\right)=\frac{Amount\;of\;drug\;in\;micelles}{Amount\;of\;added\;polymer\;and\;drug}\times 100$$

### 2.4. Cell Culture

Murine breast cancer cells (4T1) were donated by Dr. Imola Wilhelm from the Biological Research Centre (BRC) of the Hungarian Academy of Sciences from Szeged, Hungary. The 4T1 mouse triple negative breast cancer cells were cultured in RPMI 1640 (Roswell Park Memorial Institute medium) (Sigma Aldrich, R8755) supplemented with 2.4 g/L HEPES (Sigma Aldrich, H0887), 1 mM Sodium pyruvate (Sigma Aldrich, S8636), 2.5 g/L glucose (Sigma Aldrich, G7021), 2 g/L sodium bicarbonate (Sigma Aldrich, S5761), 1% ABAM (Antibiotic Antimycotic Solution) (Sigma Aldrich, A5955), and 10% fetal bovine serum (Lonza, DE14-802F, Gampel, Switzerland) in a humidified atmosphere of 5% CO_2_ at 37 °C.

### 2.5. In Vitro Cytotoxicity Assays

The cytotoxic effect of paclitaxel- and ursolic acid-loaded polymeric micelles (PTX-UA PMs) was assessed by the MTT cell-viability assay. Briefly, cells were seeded in 96-well plates at a density of 6 × 10^3^ cells per well and incubated overnight. Cells were exposed to 3 concentrations (0.01, 0.1, and 1 µM PTX) of free PTX, UA, PTX-PMs, PTX-UA-PMs, UA-PMs, and free micelles, where PMs were formulated with P2VP_55_-b-PEO_284_ and P2VP_274_-b-PEO_1406_ copolymers. The drug/copolymer ratio of polymeric micelles was 1/10. The cells were cultivated for another 24, 48 h, and 72 h, and then the medium was replaced by a medium containing 1 mg/mL MTT (3-(4,5-Dimethylthiazol-2-yl)-2,5-Diphenyltetrazolium Bromide). After a 4 h incubation, the medium with MTT was removed, and formazan crystals formed during the incubation were dissolved in MTT solution based on isopropanol according to the manufacturer’s instructions (Sigma Aldrich, Tox-1 In Vitro Toxicology Assay Kit—MTT based). The absorbance in each well was determined at 570 and 650 nm using a Tecan Infinite F200 microplate spectrophotometer (Tecan Austria GmbH, Grodig, Austria). Cell viability was calculated as the ratio of the absorbance of the wells incubated with the drug to that of the wells incubated with a culture medium.

Statistical analysis was carried out using GraphPad Prism 10 from one-way ANOVA.

## 3. Results

It was of interest to firstly analyze the colloidal characteristics, such as size, morphology, and drug encapsulation efficiencies, of the PMs. Then, the cell viability was assessed on a 4T1 mammary carcinoma mouse cell line.

### 3.1. Colloidal Characterization

**Figure 2:**
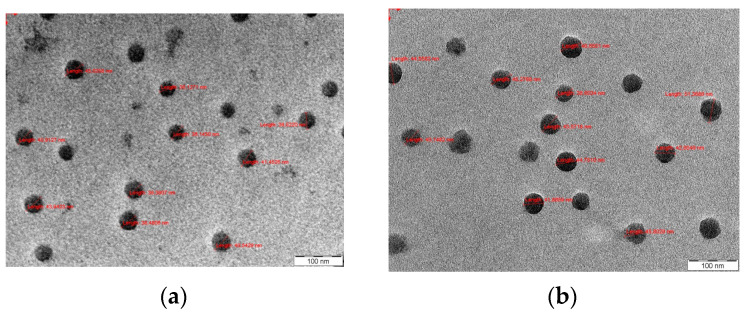
TEM photos of free PMs (**a**) and PTX-loaded PMs (**b**).

Morphology of PMs was observed by SEM, and photos are provided in Figure 2 for samples of free PMs and PTX-loaded PMs.

From Figure 2, it can be noticed that the shape of PMs is spherical and that there is no agglomeration.

Table 3 shows the colloidal data for the drug-loaded PMs at a concentration of 0.1 wt% and 25 °C obtained in PBS (pH = 7.4).

From Table 3, it is clear that the average diameter of free PMs is closely related to the molecular mass of the two copolymers [20,21]. The P2VP_274_-b-PEO_1406_ sample, having a total degree of polymerization of almost 1680, has micellar sizes almost four times larger than those of the P2VP_55_-b-PEO_284_ sample with a DPn of 339. A good correlation exists between the Z-average and Dv values with a low polydispersity index, which means that the PMs are well defined. However, a significant difference can be observed in zeta potential (ZP) values. For the P2VP_274_-b-PEO_1406_ sample, the PEO chains being much longer than those of the P2VP_55_-b-PEO_284_ sample ensures a higher protection of the micelle core formed by the pH-sensitive P2VP sequences [20,21].

As expected, the encapsulation efficiency of PTX correlated with the copolymer/drug ratio, the highest value being obtained for the highest copolymer/drug ratio (lowest drug concentration). Concerning micellar sizes, the PTX loading induces an increase of both Z-average and Dv values until a copolymer/drug ratio of 2. Moreover, the PDI values of the PTX-loaded PMs are under 0.2, which correlates with the monomodal distribution. At a high drug concentration (copolymer/drug ratio of 2), an agglomeration of micelles occurs, and two populations can be noticed on the size distribution curve.

The loading of UA led to a high increase of the micellar sizes. It is possible that some interactions, such as H bonds, occur between the PEO corona and UA leading to a decrease of the stabilizing effect of the corona and micellar aggregation, and therefore the drug was not only loaded in the micellar core but also adsorbed into the PEO corona. The modification of the ZP values can also be indicative of this adsorption.

In the case of simultaneous loading of PTX and UA, at different copolymer/drug ratios, only bimodal distributions were observed for the P2VP_55_-b-PEO_284_ copolymer. At a copolymer/drug ratio of 10, the same behavior was noticed for the P2VP_274_-b-PEO_1406_ copolymer. For both systems, the PDI values are higher than 0.4, which indicate a large polydispersity in size.

The same tendencies were noticed for the colloidal characteristics at 37 °C (Table S1 in Supporting Information).

### 3.2. FT-IR Spectroscopy

**Figure 3:**
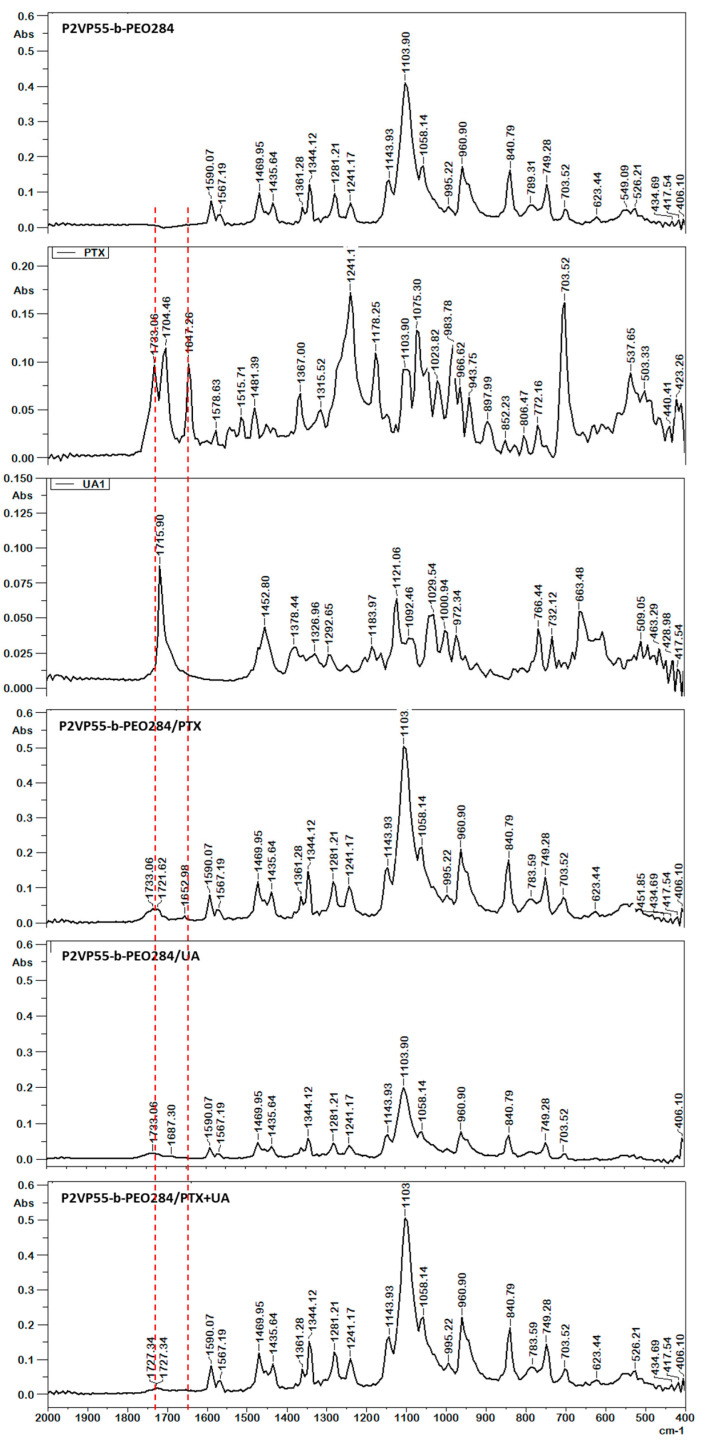
FT-IR spectra of P2VP_55_-b-PEO_284_, PTX, UA, P2VP_55_-b-PEO_284_/PTX, P2VP_55_-b-PEO_284_/UA, and P2VP_55_-b-PEO_284_/PTX + UA.

Important information about the molecular structure and the presence of loaded drugs can be obtained from FT-IR spectra. Different types of interactions between the components of a drug-loaded system induce the shift or broadening of the characteristic peaks as well as the appearance of new peaks. The potential interactions between the copolymers and both drugs were investigated by FT-IR. Figure 3 shows the FT-IR spectra for sample P2VP_55_-b-PEO_284_ in the absence and presence of PTX and UA as well as for both free drugs.

The characteristic peaks of the P2VP_55_-b-PEO_284_ copolymer appear at 3100–3600 cm^−1^ (OH stretching), 2882 cm^−1^ (CH and CH_2_), 1590 cm^−1^ (C=C stretching), 1567 cm^−1^ (C=O stretching), 1469 cm^−1^ (δCH_2_), 1435 cm^−1^ (CH stretching), 1361 cm^−1^ (δCN), 1344 cm^−1^ (CH_3_ stretching), 1281 and 1241 cm^−1^ (C-O phenol stretching), 1143 cm^−1^ (C-O-C stretching), 1103 cm^−1^ (δCN), 1058 cm^−1^ (CH_2_), 960 and 840 cm^−1^ (δCN), and 749 cm^−1^ (N-H stretching). The characteristic peaks of UA are 3554 cm^−1^ (OH); 2942 cm^−1^ (CH_3_); 1715 cm^−1^ (C=O); 1452 cm^−1^ (δCH_3_); 1378 cm^−1^ (δCH_2_); 1121 cm^−1^ (CH_3_); 1029 cm^−1^ (C-O); and 766, 732, and 663 cm^−1^ (CH) [33]. The main peaks of PTX are as follows: N-H/O-H stretching vibrations at 3479–3300 cm^−1^ and asymmetric and symmetric CH_3_ stretching vibrations at 2976–2885 cm^−1^; the peak located at 1733–1704 cm^−1^ was attributed to the C=O stretching vibration of the ester groups; the C-C stretching was located around 1647 cm^−1^; and the ester bond stretching vibrations and the C-N stretching vibrations were located at 1241 cm^−1^ and 1178 cm^−1^, respectively. Absorption at 1075, 965, and 703 was attributed to aromatic bonds [34,35]. The shifts of the characteristic peaks of both PTX and UA are evidence of their successful loading into the PMs without any chemical composition change of the drugs after loading.

### 3.3. In Vitro Cytotoxicity

Based on the encapsulation efficiencies obtained, the polymeric micelles with a drug/copolymer ratio of 1/10 were tested in vitro. For cytotoxicity testing, cells were treated for 24, 48, and 72 h with different concentrations of paclitaxel (PTX) (0.01, 0.1, and 1 µM) and ursolic acid (UA) (quantity of UA contained in PTX-UA-P2VP_55_-b-PEO_284_ and in PTX-UA-P2VP_274_-b-PEO_1406_ at each PTX concentrations), both encapsulated in polymeric micelles and in pure form. Thus, the MTT test was performed on the following experimental groups: control group (without treatment); groups treated with PTX, UA, PTX-PMs (PTX-P2VP_55_-b-PEO_284_ and PTX-P2VP_274_-b-PEO_1406_), PTX-UA-PMs (PTX-UA-P2VP_55_-b-PEO_284_ and PTX-UA-P2VP_274_-b-PEO_1406_), UA-PMs (UA- P2VP_55_-b-PEO_284_ and UA-P2VP_274_-b-PEO_1406_); and free micelles (P2VP_55_-b-PEO_284_ and P2VP_274_-b-PEO_1406_), where PMs were formulated with P2VP_55_-b-PEO_284_ and P2VP_274_-b-PEO_1406_ copolymers.

Each experimental group was tested in three different concentrations as shown in Table 4.

**Figure 4:**
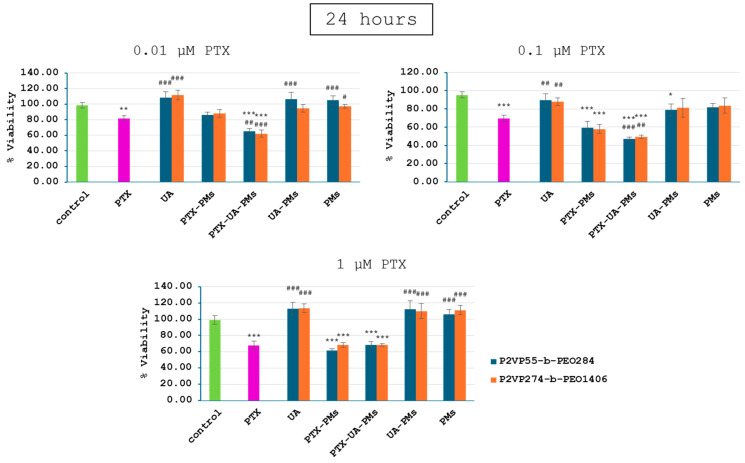
Viability of 4T1 cells following a 24 h treatment with polymeric micelles loaded with PTX and UA at the concentrations of 0.01, 0.1, and 1 μM PTX; (statistical significance: *—comparison with the control group; #—comparison with the PTX group; * *p* < 0.05; ** *p* < 0.01; *** *p* < 0.001).

**Figure 5:**
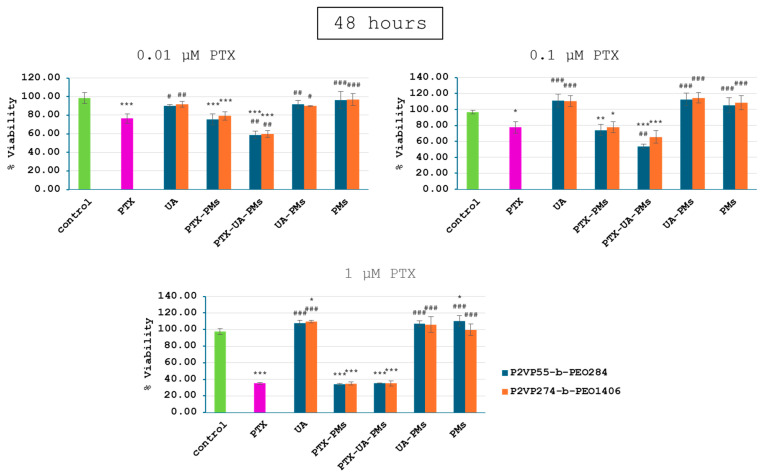
Viability of 4T1 cells following a 48 h treatment with polymeric micelles loaded with PTX and UA at the concentrations of 0.01, 0.1, and 1 μM PTX; (statistical significance: *—comparison with the control group; #—comparison with the PTX group; * *p* < 0.05; ** *p* < 0.01; *** *p* < 0.001).

**Figure 6:**
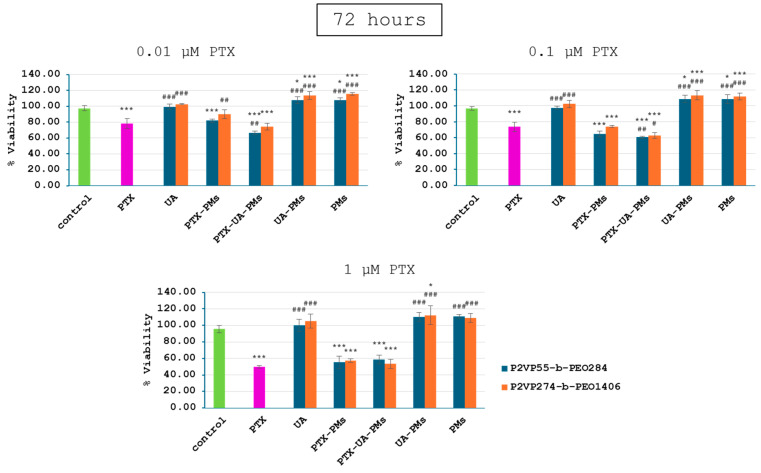
Viability of 4T1 cells following a 72 h treatment with polymeric micelles loaded with PTX and UA at the concentration of 0.01, 0.1, and 1 μM PTX; (statistical significance: *—comparison with the control group; #—comparison with the PTX group; * *p* < 0.05; *** *p* < 0.001).

Relative to the control group, 4T1 cell viability of all PTX-containing groups exhibited a dose- and time-dependent decrease manner (Figure 4, Figure 5 and Figure 6), while the addition of UA provided even greater decreases at concentrations of 0.01 and 0.1 µM PTX after 24, 48, and 72 h. Moreover, as expected, no significant differences were noticed for cells incubated with UA, as compared to the control group, due to its low concentrations.

UA-PMs and free PMs alone were not effective in inhibiting the tumor cell growth at the test concentrations. There were no significant differences in cell viability between the combination of treatments with PMs formulated with P2VP_55_-b-PEO_284_ and P2VP_274_-b-PEO_1406_.

These results suggest that polymeric micelles loaded with a combination of PTX and UA provided a significantly greater decrease in 4T1 cell viability in case of the lower concentrations, relative to PTX alone. Therefore, the synergic effect of PTX and UA was demonstrated by these in vitro tests.

## 4. Conclusions

Owing to their low critical micellar concentration, polymeric micelles (PMs) have recently attracted the interest as drug delivery systems. Of special interest were the pH-sensitive PMs as their behavior could be influenced by different pH values present in the human body. In the present study, the dual loading of paclitaxel (PTX) and ursolic acid (UA) in two pH-sensitive poly(2-vinyl pyridine)-b-poly(ethylene oxide) (P2VP-b-PEO) copolymer samples was carried out. For comparative studies, PTX and UA were loaded separately but also together in order to study their synergic effect. After the colloidal characterization of the obtained drug-loaded micellar systems, by TEM, DLS, and FT-IR, a detailed in vitro characterization was carried out. A size of around 35 nm was determined for P2VP_55_-b-PEO_284_ copolymer micelles whereas the P2VP_274_-b-PEO_1406_ sample had a micellar size of 140 nm. Moreover, it appeared that the drug loading led to a significant increase of both micellar size and PDI. As expected, PTX encapsulation efficiencies (DEEs) increase as the initial amount of drug decreased from 56 to 94%. For the dual loading of PTX and UA, the DEE increased from 54 to 64%. FT-IR spectroscopy was used to prove the drug encapsulation and the presence of some interactions between the polymer matrix and drugs. In vitro viability was studied on 4T1 mammary carcinoma mouse cells as a function of time and concentration. No significant differences were noticed between the two PM samples, which indicate that their sizes have no effect on the cell viability. UA-loaded PMs and free PMs were not effective in inhibiting the tumor cell growth at the tested concentrations. For PTX-loaded PMs and PTX/UA-loaded PMs, the cell viability was comparable to the values obtained for PTX alone, but the concentration of loaded drugs was much smaller. Moreover, a synergic effect was noticed for the dual PTX/UA-loaded PMs. The obtained in vitro results demonstrate that the dual PTX/UA-loaded PMs could be of further use for in vivo tests on animals.

## Figures and Tables

**Table 1:** Block copolymer molecular characteristics.

Samples	M_n_ (P2VP) (g/mol) ^a^	M_n_ (PEO) (g/mol) ^a^	M_n_ (Copolymer) (g/mol)	M_w_/M_n_ (Copolymer) ^a^
P2VP_55_-b-PEO_284_	5800	12,500	18,300	1.11
P2VP_274_-b-PEO_1406_	28,800	61,900	90,700	1.29

^a^ by SEC data using PS standards.

**Table 2:** Experimental parameters for the preparation of drug-loaded micelles.

Sample	Drug	Cop/Drug Ratio	Quantity (mg)		
			m (Copolymer)	m (PTX)	m (UA)
P2VP_55_-b-PEO_284_	PTX	20/1	100	5	
		10/1	100	10	
		5/1	100	20	
		2/1	100	50	
	PTX + UA	10/1	100	5	5
		5/1	100	10	10
		2/1	100	25	25
	UA	10/1	100		10
	Free PMs		100		
P2VP_274_-b-PEO_1406_	PTX	10/1	100	10	
	PTX + UA	10/1	100	5	5
	UA	10/1	100		10
	Free PMs		100		

**Table 3:** Colloidal data of drug-loaded PMs at a concentration of 0.1 wt% and 25 °C obtained in PBS (pH = 7.4).

Sample		Copolymer/ Drug Ratio (mg/mg)	DEE (%)	DLE (%)	Z-Average (nm)	Dv (nm)	PDI	ZP (mV)
P2VP_55_-b-PEO_284_	Free PMs	-	-	-	43.1	34.5	0.261	−15.5
	PTX	20	94.0	4.5	45.0	38.1	0.143	−17.2
		10	91.6	8.3	51.8	45.7	0.107	−18.2
		5	64.2	10.7	71.1	59.8	0.122	−17.6
		2	56.0	18.7	n.a	43.1–85% 312–15%	n.a	−3.3
	UA	10	92.1	8.3	348.9	477.3	0.228	−1.5
	PTX + UA	10	64.7	5.9	223.2	584–27% 41–73%	0.624	−4.3
		5	65.0	8.4	305.7	654–66% 50–34%	0.440	−2.1
		2	53.8	17.9	612.4	1150–96% 168–4%	0.356	−1.9
P2VP_274_-b-PEO_1406_	Free PMs	-	-	-	140.5	141.7	0.044	−10.1
	PTX	10	62.3	5.5	1761.3	1178–95% 135–5%	0.859	−1.7
	UA		61.9	5.4	309.8	n.d	0.453	−1.4
	PTX + UA		71.2	3.2	1625.3	1596–95% 231–5%	0.657	−1.5

**Table 4:** PTX and UA (quantity of UA contained in PTX-UA-P2VP_55_-b-PEO_284_ and in PTX-UA- P2VP_274_-b-PEO_1406_ at each PTX concentration) concentrations used for the MTT test.

	PTX	UA	
		P2VP_55_-b-PEO_284_	P2VP_274_-b-PEO_1406_
C1	0.01 µM PTX	34.72 nM UA	20.65 nM UA
C2	0.1 µM PTX	347.2 nM UA	206.5 nM UA
C3	1 µM PTX	3472 nM UA	2065 nM UA

## Data Availability

Data are contained within the article.

## Supplementary Materials

The following supporting information can be downloaded at: https://www.mdpi.com/article/10.3390/polym16091189/s1, Table S1: Colloidal data of drug-loaded PMs at a concentration of 0.1 wt% and 37 °C obtained in PBS (pH = 7.4).
