# Supplementary material for: Colloidal and Biological Characterization of Dual Drug-Loaded Smart Micellar Systems
Source: Polymers (Basel). 2024 Apr 24;16(9):1189. doi: 10.3390/polym16091189 (PMC11085147; doi:10.3390/polym16091189)
Supplement: Supplementary file 1 [file polymers-16-01189-s001.zip › polymers-2963900-supplementary.pdf]

Supporting Information

Colloidal and biological characterization of dual drug-loaded smart micellar systems

Hildegard Herman[1], Delia. M. Rata[2], Anca N. Cadinoiu[2], Leonard I. Atanase[2,3,*], Anca Hermenean[1]

[1] “Aurel Ardelean” Institute of Life Sciences, Vasile Goldis Western University, Rebreanu Street, No. 86, 310414 Arad, Romania; H.H - hildegard.i.herman@gmail.com; A.H. - anca.hermenean@gmail.com

[2] Faculty of Medicine, “Apollonia” University of Iasi, Pacurari Street, No. 11, 700511 Iasi, Romania; D.M.R. - iureadeliamihaela@yahoo.com; A.N.C. - jancaniculina@yahoo.com; L.I.A. – leonard.atanase@yahoo.com

[3] Academy of Romanian Scientists, 050045 Bucharest, Romania

Table S1: Colloidal data of drug-loaded PMs at a concentration of 0.1 wt% and 37°C obtained in PBS (pH = 7.4).

Sample		Copolymer/Drug Ratio (mg/mg)	Z-average (nm)	Dv (nm)	PDI
JPL 12	Free PMs	-	41.6	34.3	0.251
	PTX	20	44.5	38.0	0.199
		10	51.3	44.2	0.132
		5	68.6	58.6	0.108
		2	n.d		n.d
	UA	10	353.7	507.8	0.256
	PTX+ UA	10	226.4	648-33% 44.5-67%	0.667
		5	319.4	643-65% 55.7-35%	0.415
		2	618.6	1102-96% 111-4%	0.360
JPL7	Free PMs	-	140.4	141.5	0.041
	PTX	10	1851	1202-95% 131-5%	0.786
	UA		312.4	n.d	0.435

PTX+ UA		1648	1458-95% 290-5%	0.732
